# Supplementary material for: The many faces of compensation: The similarities and differences between social and facial models of perception
Source: PLoS One. 2024 Feb 23;19(2):e0297887. doi: 10.1371/journal.pone.0297887 (PMC10890726; doi:10.1371/journal.pone.0297887)
Supplement: Attachment — Submitted filename: response_to_reviewers.docx [file pone.0297887.s001.docx]

**Comments to the Author**

1. If the authors have adequately addressed your comments raised in a previous round of review and you feel that this manuscript is now acceptable for publication, you may indicate that here to bypass the “Comments to the Author” section, enter your conflict of interest statement in the “Confidential to Editor” section, and submit your "Accept" recommendation.

Reviewer #1: All comments have been addressed

Reviewer #2: (No Response)

2. Is the manuscript technically sound, and do the data support the conclusions?

The manuscript must describe a technically sound piece of scientific research with data that supports the conclusions. Experiments must have been conducted rigorously, with appropriate controls, replication, and sample sizes. The conclusions must be drawn appropriately based on the data presented.

Reviewer #1: Yes

Reviewer #2: Yes

3. Has the statistical analysis been performed appropriately and rigorously?

Reviewer #1: I Don't Know

Reviewer #2: Yes

4. Have the authors made all data underlying the findings in their manuscript fully available?

The PLOS Data policy requires authors to make all data underlying the findings described in their manuscript fully available without restriction, with rare exception (please refer to the Data Availability Statement in the manuscript PDF file). The data should be provided as part of the manuscript or its supporting information, or deposited to a public repository. For example, in addition to summary statistics, the data points behind means, medians and variance measures should be available. If there are restrictions on publicly sharing data—e.g. participant privacy or use of data from a third party—those must be specified.

Reviewer #1: Yes

Reviewer #2: Yes

5. Is the manuscript presented in an intelligible fashion and written in standard English?

PLOS ONE does not copyedit accepted manuscripts, so the language in submitted articles must be clear, correct, and unambiguous. Any typographical or grammatical errors should be corrected at revision, so please note any specific errors here.

Reviewer #1: Yes

Reviewer #2: Yes

6. Review Comments to the Author

Please use the space provided to explain your answers to the questions above. You may also include additional comments for the author, including concerns about dual publication, research ethics, or publication ethics. (Please upload your review as an attachment if it exceeds 20,000 characters)

Reviewer #1: (No Response)

Reviewer #2: This manuscript tackles an important topic in the domain of social psychology, focusing on the compensation effect in visual impressions, which appears to be a novel angle.

Areas for Improvement:

Clarify Terminology: The use of jargon and specialized terms (e.g., "CI") without adequate initial definition can be a barrier for readers unfamiliar with the specific nuances of the topic. Ensure that all terms are defined upon first mention.

Streamline Content: Some sections have redundancy. Reducing repeated content can make the manuscript more concise and engaging.

Enhance Accessibility: The manuscript, especially the discussion, is dense in places. Consider simplifying complex sentences and breaking down intricate ideas for broader readership.

Citation Format: There are placeholders for citations throughout. Ensure they are replaced with proper references in a consistent format.

Enhanced Structure: Consider using subheadings more extensively to provide a clearer roadmap for readers, especially in the methods and discussion sections.

Ethical Considerations: While it's not clear from the presented sections, make sure ethical considerations related to the study's execution are explicitly mentioned elsewhere in the manuscript.

The manuscript presents a well-researched, detailed examination of the compensation effect in visual impressions. Given its novel approach and thorough exploration, it holds significant value to the field. With some refinements, particularly in enhancing clarity, streamlining content, and ensuring all terms and concepts are defined, the manuscript appears to be of publishable quality. The considerations highlighted above should strengthen the manuscript, making it even more compelling for publication.

*Authors’ response:*

*We thank Reviewer #2 for the valuable suggestions to strengthen our manuscript.*

*We enhanced the manuscript's clarity and readability by carefully defining specialized terms at their first occurrence (i.e., RC & CI) and on multiple occasions throughout the manuscript. We made sure to condense overlapping sections without omitting key information. Furthermore, we also simplified the discussion to make the manuscript more concise and engaging.*

*Following PLOS guidelines, we thoroughly implemented the “Vancouver” reference style. Specifically, references are listed at the end of the manuscript and numbered in the order that they appear in the text. In the text, we cite the reference number in square brackets.*

*Regarding the Ethical consideration, we slightly refined the Ethical statement section. Additionally, we made sure to mention in the discussion section that we adhered to the Ethical guidelines.*

7. PLOS authors have the option to publish the peer review history of their article (what does this mean?). If published, this will include your full peer review and any attached files.


If you choose “no”, your identity will remain anonymous but your review may still be made public.


Do you want your identity to be public for this peer review? For information about this choice, including consent withdrawal, please see our Privacy Policy.

Reviewer #1: No

Reviewer #2: No
